# Cryo-EM structure of the diapause chaperone artemin

**DOI:** 10.3389/fmolb.2022.998562

**Published:** 2022-11-28

**Authors:** Amar D. Parvate, Samantha M. Powell, Jory T. Brookreson, Trevor H. Moser, Irina V. Novikova, Mowei Zhou, James E. Evans

**Affiliations:** ^1^ Pacific Northwest National Laboratory, Environmental Molecular Sciences Laboratory, Richland, WA, United States; ^2^ Washington State University Pullman, School of Biological Sciences, Pullman, WA, United States

**Keywords:** artemin, cryo-EM, chaperone, native MS, cell-free expression

## Abstract

The protein artemin acts as both an RNA and protein chaperone and constitutes over 10% of all protein in *Artemia* cysts during diapause. However, its mechanistic details remain elusive since no high-resolution structure of artemin exists. Here we report the full-length structure of artemin at 2.04 Å resolution. The cryo-EM map contains density for an intramolecular disulfide bond between Cys22-Cys61 and resolves the entire C-terminus extending into the core of the assembled protein cage but in a different configuration than previously hypothesized with molecular modeling. We also provide data supporting the role of C-terminal helix F towards stabilizing the dimer form that is believed to be important for its chaperoning activity. We were able to destabilize this effect by placing a tag at the C-terminus to fully pack the internal cavity and cause limited steric hindrance.

## 1 Introduction

Species of the brine shrimp *Artemia* are found across North, Central and South America and inhabit some of the most challenging environments (Clegg and Gajardo, 2009). The key to surviving such harsh conditions has been tracked to the brine shrimp’s ability as a cyst to enter a state of metabolic hypoactivity called diapause. In this state, the cyst can survive desiccation, high and low temperatures, radiation, and years of anoxia (Takalloo et al., 2020a). A complement of stress tolerance proteins have been reported in *Artemia* during diapause including p26, artemin and hsc70 (Clegg and Gajardo, 2009). Of this group, artemin is particularly interesting due to evidence that it acts as both a protein and RNA chaperone (Warner et al., 2004). Excluding the yolk, artemin can constitute 10–15% of the total protein content of cysts in diapause (Hassani and Sajedi, 2013). Additionally, *in vitro* studies have shown artemin to be highly thermostable and to demonstrate chaperone-like activity under prime stressors such as exposure to heat, H_2_O_2_, or both, and also exposure to cold (Hassani and Sajedi, 2013; Takalloo et al., 2016; Takalloo et al., 2017).

While artemin is a ferritin homolog, its differences, rather than similarities, to ferritins shed more light on its role as a chaperone. Artemin monomers are 229 amino-acid residues long with a molecular mass of 26 kDa and form 24mers with a mass of ∼624 kDa. The artemin monomer is 45–50 residues longer than most ferritins, even though they form oligomers of similar dimensions and symmetry (Chen et al., 2003). Unlike ferritins whose job is to sequester iron, artemin is unable to bind iron due to naturally modified regions of the ferroxidase center, iron nucleation center and 3-fold channel. Additionally, artemin is a thiol rich molecule with nine free thiols and one thiol involved in a disulfide bond (Hu et al., 2011). Importantly, several biochemical studies point to the chaperone activity of artemin being regulated by a redox switch courtesy of the thiols (Mosaddegh et al., 2018) as well as its C-terminus which diverges considerably from ferritins (Rasti et al., 2009).

Molecular chaperones are broadly divided into holdases and foldases. Foldases are ATP dependent chaperones which actively support folding of proteins in the right conformation. Examples from bacteria include the GroEL/GroES or the DnaK/DnaJ/GrpE system, while the Hsp60/70/90 family of chaperones are examples of foldases in mammalian systems (Graf and Jakob, 2002; Hoffmann et al., 2004). Holdases, also called small heat shock proteins (sHSPs) are ATP-independent chaperones that include the bacterial protein Hsp33, Get3 in yeast and its human analog TRC40. Biochemical reports suggest that many holdases are regulated and reversibly activated *via* a redox switch. Brine shrimp have been reported to have their own complement of holdase (p26) and foldase (Hsc70) chaperones along with artemin (Clegg and Gajardo, 2009). Foldases seem to prefer higher molecular weight assemblies (GroEL) while holdases typically exist as monomers or dimers of 10–40 kDa (Niforou et al., 2014) and dimerize on stress dependent activation. Artemin is believed to act as a protein and RNA chaperone and shares a lower molecular weight characteristic of holdases, but it exists as a 24mer and upon exposure to stress, releases oligo n-mers of which dimers are most abundant. Artemin also lacks an α-crystallin domain which is otherwise ubiquitous in sHSPs. Interestingly, artemin is modeled to form head to tail dimers like 2-Cys perioxiredoxins (2-Cys Prxs)—another redox mediated holdase (Kumsta & Jakob, 2009) which forms higher molecular weight assemblies (10 or 12 mers) upon exposure to increasing amount of stress. The most drastic difference between artemin and other holdases is the irreversible structural changes that occur on exposure to stresses like heat or H_2_O_2_ whereas other redox regulated holdases are reversible (Takalloo et al., 2020b). Artemin therefore appears to be a holdase-like chaperone with unique properties though additional mechanistic details remain elusive due to a lack of experimentally derived structure.

All prior structural hypotheses for artemin function were based upon computationally derived homology models using apoferritin as a template (Rasti et al., 2012). The homology models indicated that the core of artemin has a similar fold to apoferritin, including the five core ferritin helices (A-E) and the hydrophobic loop L. However, the first twenty N-terminal residues of artemin were suggested to exist as flexible loops directed outwards and solvent exposed, while the C-terminal residues were predicted to curve inwards into the cavity of the artemin (Shirzad et al., 2011). Other than *in silico* data suggesting that the C-terminus completely fills the central cavity of artemin, there was no consensus in prior literature on the fold or secondary structure of the C-terminus despite this region having significant roles in chaperone activity. Additionally, none of the prior reported homology models are currently publicly available as they were not posted to sustained repositories and this makes continued studies difficult.

Based on homology models and biochemical data, a mechanism of action for the chaperoning activity of artemin has been suggested to rely on the activation through a cysteine redox switch in response to environmental stressors. This leads to the breakdown of the 24mer into smaller oligomers, of which, dimers are believed to be most abundant and to be the functional chaperone (Takalloo et al., 2020b). The stable dimer putatively interacts with the target protein through the C-terminal helices to stabilize the target protein and prevent either denaturation, unfolding, or both. Chaperone activity has been observed to stay at peak levels under multiple conditions such as between 25–50°C, in presence of 40–100 mM hydrogen peroxide, and following exposure to cold or hypersaline environments (MacRae, 2016; Takalloo et al., 2016). Several factors have been proposed to play an essential role in artemin chaperoning activity including the number of free and solvent exposed thiols, existence of exposed hydrophobic surfaces and also the local environment of Trp, Tyr and His residues (Hu et al., 2011; Mosaddegh et al., 2018). However, the absence of a high-resolution structure of artemin has led to competing theories for artemin’s mechanism of action based on prior homology models and left the ultimate structural details of the protein elusive.

Here we used an integrative approach combining cell-free expression, cryo-electron microscopy (cryo-EM), and native mass spectrometry to determine the atomic structure of artemin. We provide a structure of full-length artemin at 2.04 Å using single particle cryo-EM coupled with cell-free expression. Native mass spectrometry (MS) was used to confirm the molecular weight of all species and probe the stability of artemin dimerization since the dimer form is believed to be the functional subunit while chaperoning.

## 2 Materials and methods

### 2.1 Protein expression and purification

DNA plasmids for artemin were prepared by Genscript using their custom gene synthesis and cloning services. Obtained DNA templates (pEU_artemin_6His and pEU_3XF_artemin) were used in the cell-free gene expression and protein purification by Protemist DTII, an automated protein synthesizer from CellFree Sciences, using well-established in-house protocols (Novikova et al., 2018) and manufacturer’s guidelines. Supplementary Table S1 shows the amino acid sequences for all clones. For 3XFlag-based purification on the Protemist DTII, 800 µl of ANTI-Flag M2 Affinity gel (Sigma, A2220) was used per 6-ml translation reaction. For his-tag based purification, 800 µl of Ni Sepharose 6 fast flow (Sigma, GE17-5318-01) was used per 6-ml translation reaction. Additionally, in all reactions, SUB-AMIX buffer was supplemented with protease inhibitor cocktail (Sigma Aldrich, #539137) with the buffer to cocktail ratio of 100:1 (v/v). For the expression of fluorophore-labeled proteins, the translation mixture was supplemented with FluoroTect GreenLys reagent (Promega). Purified samples were washed with TBS (50 mM Tris and 150 mM NaCl, pH 7.5) buffer and concentrated in a pre-chilled centrifuge at 15,000×g to a final volume of 500 µL using a 0.5 ml 10 kDa MWCO Amicon spin column.

Concentrated proteins were further loaded onto an AKTA Pure FPLC system stored at 4°C using either a Superose 6 Increase 10/300 or Superdex 200 Increase 10/300 column. Collected fractions corresponding to the protein peak on the AKTA SEC trace were combined and concentrated using 10 kDa MWCO Amicon spin columns. Protein purity was verified by SDS and Native PAGE.

### 2.2 Cryo-EM sample preparation and single particle data collection

3 µL of apoferritin or artemin solution at 0.2–1.5 mg/ml were loaded on to glow discharged Quantifoil grids (200 mesh R2/1 or 300 mesh R1.2./1.3). Grids were blotted for 1.5–3.5 s and plunge frozen in liquid ethane on a Leica EM GP2. Grids were stored in liquid nitrogen until further use. For screening and data collection, grids were loaded on a 300 keV Titan Krios G3i (Thermo Fisher) and all datasets were collected using the standard EPU software along with K3 direct electron detector and a Bioquantum energy filter (Gatan Inc.) with 20 eV slit. Movies were collected at ×130,000 magnification in super resolution mode resulting in a pixel size of 0.3398 Å or at 215,000x in conventional counting mode and 1x binning resulting in a pixel size of 0.4108 Å. Movies were collected at a total dose ranging from 40 to 59 e^−^/Å^2^, with 0.5–1.8 s exposures, and a defocus range of −0.3 to −1.3 µm. Details of data collection are mentioned in Supplementary Table S2.

### 2.3 Image processing

All movies were processed using cryoSPARC Live and cryoSPARC (Punjani et al., 2017). Motion correction and CTF estimation were performed using default parameters and initial particle extraction used the built-in *blob picker* with a box size of 400 or 800 pixels (Rubinstein and Brubaker, 2015). Details about particle numbers at each step are listed in Supplementary Table S2. Initial subsets of particles were subjected to reference free 2D classification before discreet and diverse classes were chosen to re-extract particles using template picking. Multiple rounds of classification were performed to exclude junk and non-homogenous classes. Ab-initio models were generated using a subset of these particles and C1 symmetry. The entire particle set was refined in 3D against ab-initio models without symmetry. Octahedral symmetry was imposed in subsequent rounds of refinement. Per particle local CTF refinement was performed before the final round of homogenous refinement. (Terwilliger et al., 2018). Resolution of the final map was estimated using the gold standard at 0.143 FSC. Maps were visualized using UCSF Chimera (Pettersen et al., 2004) and have been deposited in the EMDB (Flag-artemin = EMD-24706, artemin-His = EMD-24707, apoferritin = EMD-24145).

### 2.4 Modelling

The initial homology model for artemin was generated using (Zimmermann et al., 2018) HHPRED and MODELLER (Webb and Sali, 2016) based on the top 15 aligned sequences to known ferritin structures. Additional models were also generated using AlphaFold2 and RosettaFold. All models were initially docked into the raw artemin map using *Dock in Map* (Afonine et al., 2018). The initial model from MODELLER had the best initial score following docking and was therefore used for all following steps. An initial round of refinement with Phenix *Real-space Refinement* was carried out on the docked model. The initial docked model was missing N-terminal residues 1–25 and C-terminal residues 202–229. To improve the clarity of the density map of Flag-artemin, the *Autosharpen map* tool in Phenix (Terwilliger et al., 2018) was used. Using the sharpened map, iterations of model building in COOT (Emsley et al., 2010) and refinement in Phenix were executed, and the entirety of the C-terminus, and residues 22–25 were built into the model. Model validation of the monomer and dimer was performed using Molprobity (Williams et al., 2018). Using the symmetry file generated by *Map Symmetry* in Phenix, the full artemin 24mer was modeled into the map. The final model of Flag-artemin was deposited to the PDB (PDB: 7RVB).

### 2.5 Native mass spectrometry

Protein samples were dialyzed overnight in 200 mM ammonium acetate using 96-well Microdialysis units (10 k MWCO, Pierce). If further salt removal was needed, additional buffer exchange was performed using Zeba Spin Desalting Columns (7 k MWCO, 75 μl, Thermo Fisher). Final concentrations used for native MS were 1–2 µM. All native MS data was acquired on a Waters Synapt G2s-i ion mobility time-of-flight mass spectrometer. Nanoelectrospray voltage (0.6–0.8 kV) was applied through a Pt wire inserted into hand-pulled borosilicate glass capillaries (Sutter Instrument) which contained the protein solution. To filter the artemin 24mer from low *m/z* species prior to collision induced dissociation (CID), a manual fixed quad profile of 10,000 was used. MassLynx v4.1 (Waters) was used to manually analyze spectra and mass deconvolution was performed using UniDec version 4.3.0 (Marty et al., 2015).

## 3 Results

### 3.1 Artemin single particle cryo-EM map revealed a unique central cavity

**FIGURE 1:**
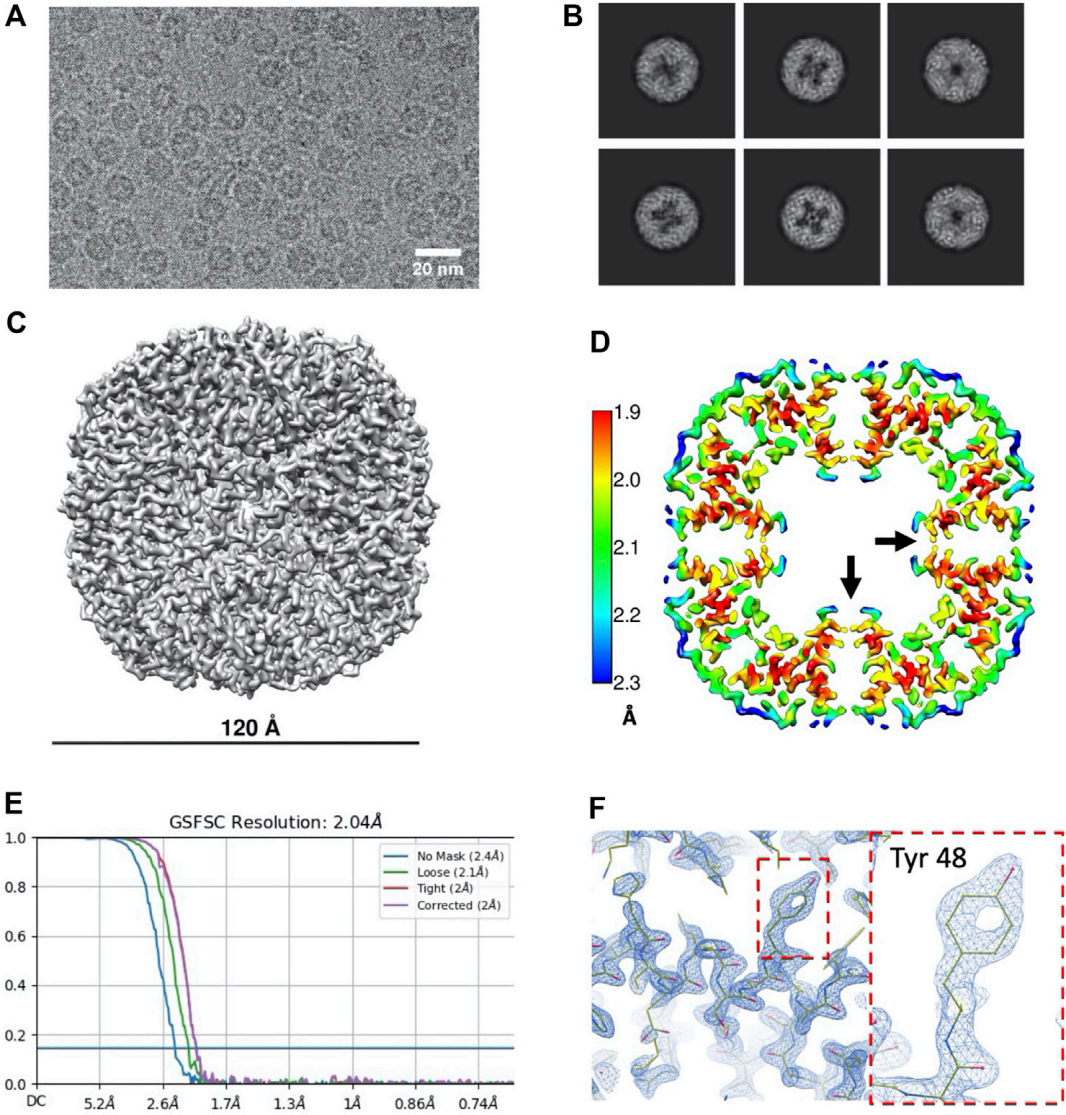
Data processing results for Flag-artemin. **(A)** Representative micrograph of artemin showing overall dimensions similar to apoferritin but the central cavity is partially filled with density **(B)** 2D classes showing that while the C-terminus of the monomer does point inwards from the shell, it does not fully fill the central cavity. **(C)** Cryo-EM map of Flag-artemin with ∼120 Å outer diameter. **(D)** Thin virtual slice through a resolution heat map showing the C-terminal alpha helices pointing inwards (black arrow) into the cavity. **(E)** Resolution estimated by gold standard at 2.04 Å at 0.143 FSC. **(F)** Fit of the atomic model in the sharpened map using Coot. Dashed boundary highlights are zoomed-in with the inset showing the detailed fit for Tyr 48.

Ever since artemin’s first report in 1980 (Slobin, 1980), a growing body of reports have elucidated the role of artemin as a molecular chaperone (Shirzad et al., 2011; Hassani and Sajedi, 2013; Takalloo et al., 2016), but structural information about the protein has been limited to *in silico* modelling and some spectroscopic studies to date (Takalloo et al., 2020a). We sought to determine the full-length structure of artemin experimentally using single particle cryo-EM. To generate the protein sample, we employed cell-free protein expression (coupled transcription and translation reactions in a test tube) and purification protocols well-established in-house (Novikova et al., 2018; Novikova et al., 2021). Using an N-terminal tagged 3XFLAG artemin construct (Flag-artemin) from *A. fransciscina* we obtained 250 µg of protein within 3 days of receiving the synthesized clone, which was sufficient quantity and purity for our needs. To obtain even more homogenous sample, artemin was further purified using size exclusion chromatography (Supplementary Figures S1, S2) prior to plunge freezing on cryo-EM grids followed by single particle screening and data collection. Total timeframe from receiving the synthesized clone through cell-free expression, purification, cryo-EM screening, data collection and 3D refinement was only 9 days. The motion corrected cryo-EM images showed rosette-like artemin particles with a diameter of ∼120 Å (Figure 1A). However, the central cavity of artemin is not completely filled as suggested by previous modelling studies, as evident in the raw images as well as 2D class averages (Figure 1B). While no symmetry was applied for 3D ab-initio model generation and initial 3D refinement, those results clearly revealed an octahedral symmetry which matched with the expected 24mer assembly state for artemin. Therefore, octahedral symmetry was imposed in subsequent steps of 3D reconstruction and refinement and led to a final map at 2.04 Å at 0.143 FSC (Figures 1C–F **).**

**FIGURE 2:**
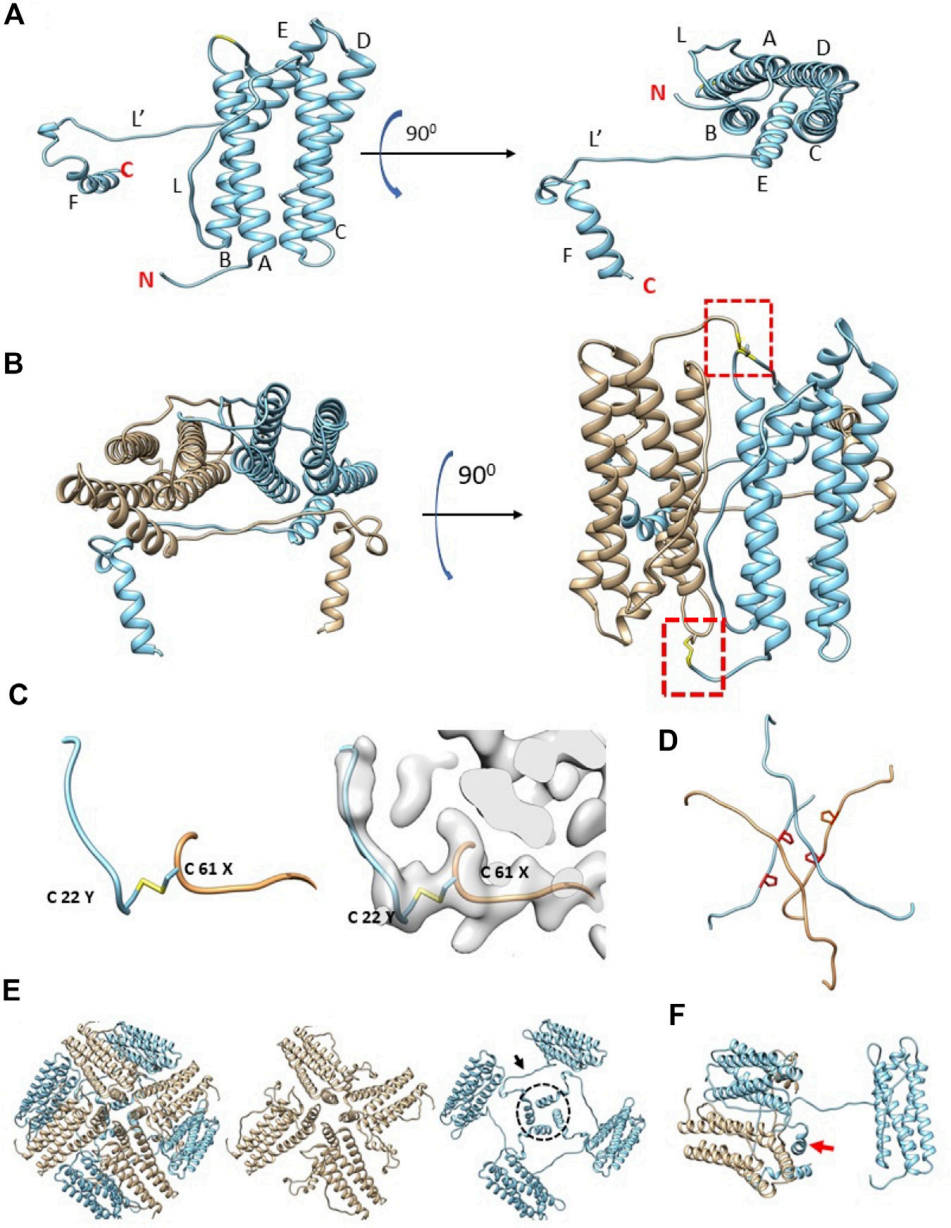
Structural organization of artemin. **(A)** Artemin monomer with helices A-F and loops L and L′ annotated. The extra length of helix E for artemin compared to apoferritin helps position loop L′ to run along the inside of the core shell of artemin before helix F turns inward into the artemin cavity. N and C termini are indicated in red. **(B)** Artemin dimer with antiparallel monomers colored separately (tan vs. sky blue). Dashed border indicates area of the Cys22-Cys61 disulfide bond. **(C)** A zoomed in view of the region in the dashed boundary in **(B)** shows the Cys22-Cys61 disulfide bond, with and without the map density. **(D)** The L and L′ loops from respective monomers forming the hashtag arrangement. Side chains of Pro198 and Pro201 are shown in red for each loop. **(E)** Four artemin dimers around a 4-fold axis. The conventional 4-fold axis has loops from monomers containing Cys172 (tan) similar to apoferritin arrangement. In addition, the complementary monomers (sky blue) in apoferritin have a second arrangement (dashed circle) where loop L’ (arrow) and helix F contact extend towards the neighboring dimer. **(F)** Helix F (red arrow) interacts with both monomers from the neighboring dimer.

Prior sequence alignment, homology modeling and molecular dynamics studies had predicted that the structure of artemin would be similar to apoferritin (Rasti et al., 2012) with the exception of the artemin C-terminus filling the inner cavity. After fitting an initial homology model of artemin into the cryo-EM density map, the model was corrected and refined with a combination of COOT and Phenix (Supplementary Tables S2, S3). In total, all residues for artemin except the first 21 N-terminal residues were modeled. The fit confirms that the overall organization of artemin is analogous to apoferritin (Supplementary Figure S2 and Supplementary Table S4) with residues 30–173 of artemin forming a similar shell structure as apoferritin (PDB: 4V1W) comprised of 5 α-helices (A-E) and one long disordered loop (L). Major differences arise due to artemin having a 28 residue long disordered N-terminus region as well as an additional helix (F) and a second long disordered loop (L′) (Figure 2A, Supplementary Figure S3). Importantly, this experimentally determined 3D structure of artemin has density corresponding to the entire C-terminus and clearly shows that the internal cavity is not completely filled in contrast to prior *in silico* models. Although prior molecular dynamics simulation studies suggested that the C-terminus of artemin forms α-helices that extend inwards into the cavity of the molecule to fill the space, our cryo-EM map of Flag-artemin and the corresponding fitted atomic model clearly show that the C-terminal residues do ultimately turn inwards, but they first hug the inner surface of the core artemin shell before extending only partly into the artemin cavity. Interestingly, the unique loop L′ of artemin is oriented orthogonal to loop L and the apoferritin-like 4-helix bundle at the 4-fold channel. Artemin’s loop L′ contains Pro198 and Pro201 which potentially prevents this region from forming into a helical conformation and helps favor the interaction with the inner surface of the shell. A third proline in the C-terminus (Pro213) provides a kink that results in the C-terminal helix F (aa 216–229) turning into the cavity of artemin (Figures 2A,B).

Previous publications noted that artemin only retains one of seven conserved residues related to ferritin ferroxidase activity, only one of six conserved residues for the 3-fold channel and none of the four conserved residues for iron nucleation (Rasti et al., 2009). The atomic model based on our cryo-EM map clearly confirms a lack of a charged 3-fold channel. Interestingly, the residues typically associated with iron nucleation in ferritin are directly occluded in the experimentally determined artemin atomic model due to the presence of the extra loop L’. Iron nucleation in ferritin is achieved by four glutamate residues Glu53, Glu56, Glu57, and Glu60 (Rasti et al., 2009), but those same residues in artemin are Trp73, His76, Val77, and Gln80 (Supplementary Figure S2A). While these amino acid differences for artemin relative to apoferritin would prevent nucleation simply due to the change in electrostatics, this change also facilitates the interaction with loop L′ by removing the highly negatively charged four glutamate residues in an 8-residue span. Thus, the presence of loop L’ also prevents iron nucleation. Other amino acid differences between artemin and ferritin show a general change in electrostatic surface potential even though the Coulombic surface map looks very similar (Supplementary Figure S4).

Previous biochemical studies combined with homology modeling have indicated that several conserved cysteines in artemin are essential for structural integrity and the putative chaperone activity of artemin (Hu et al., 2011), while the C-terminus was found to be important for the overall thermostability of artemin. Additionally, recent reports have identified the artemin dimer as the putative unit that has chaperone activity. In our experimentally derived model, the artemin dimer is oriented similarly to an apoferritin dimer (Figure 2B) and a disulfide bridge exists between Cys61 and Cys22 of neighboring opposite facing monomers. This confirms the presence of two disulfide bridges per dimer (Figure 2C) which is in line with previous homology modeling (Hu et al., 2011) and biochemical studies (Mosaddegh et al., 2018) that identified structural, but not functional, artemin destabilization at high temperatures when either or both of these Cys residues were modified. None of the other eight cysteines are observed to be involved in disulfide bridges likely because all are surface exposed.

The overall octahedral symmetry of artemin shows an extra stabilizing interaction relative to apoferritin where the L′ loops or two monomers form a hashtag arrangement that connects the 4-helix bundles from each monomer in addition to the ferritin-like L loop interaction between two monomers at the outer surface (Figure 2D). Somewhat surprisingly, loop L′ and helix F extend and contact neighboring dimers which differs from all prior reported homology modeling efforts. This results in helix F from each monomer forming a second 4-helix bundle toward the center of the complex (Figures 2D–F). Helix E from one dimer interacts with helices from three neighboring dimers around a 4-fold axis like apoferritin. For example, the ferritin monomer would contact chains at the 2- (dimer), 3- and 4-fold interfaces. In addition, near the 4-fold interface in artemin, helix F from neighboring chains form a second interaction facilitated by the respective antiparallel loops L’ (Figures 2A,E) These additional inter- and intradimer interactions resulting from loop L’ and helix F may contribute to the significant thermal stability of artemin.

**FIGURE 3:**
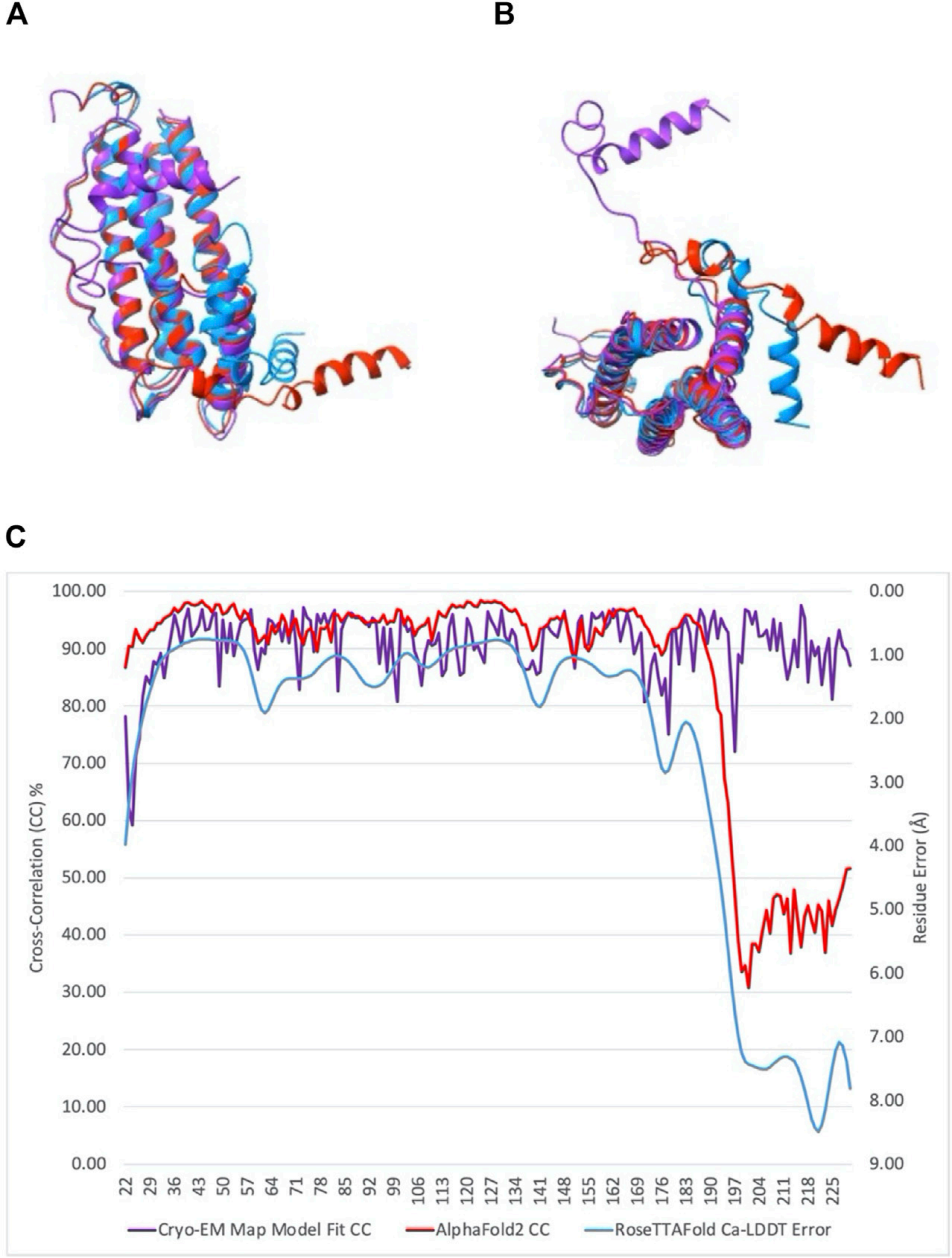
Comparison of the structure of artemin monomer with models obtained from AlphaFold2 and RoseTTAFold. **(A)** Final atomic model of artemin monomer calculated by direct refinement and fitting within our cryo-EM map (purple) overlaid atop models from AlphaFold2 (red) and RoseTTAFold (blue). **(B)** 90-degree rotation of models in **(A)**. **(C)** Plot comparing correlation scores of cryo-EM map/model fit and AlphaFold2 confidence scores per residue plotted against left axis, and RoseTTAFold per residue error in Å plotted against right axis. Colors for plots match the plots of the overlays in **(A,B)**.

Models of artemin created with AlphaFold2 and RosettaFold (Figure 3) (Baek et al., 2021; Jumper et al., 2021) show a similar fold for the core region (as expected due to high homology with ferritin), but they fail to capture the C-terminal loop L’ and full helix F positioning. The hashtag arrangement and the interactions of helix F with the neighboring dimer may be important in the context of the 24mer structure. They may rearrange when exposed to temperature or oxidation when in their dimer or monomer state and these may be what AlphaFold2 and RosettaFold are predicting. Though further experimental work will need to be performed to validate those models.

### 3.2 Structural perturbation identified potential features that affect artemin’s stability

The C-terminal helices of artemin are implicated in chaperone activities (Shirzad et al., 2011) and previous homology models suggested that the C-terminus fully packs the inside core of artemin. While our cryo-EM map of Flag-artemin clearly shows that the native C-terminus does not fully pack the interior of the artemin octahedral complex, we wondered what would happen if we intentionally filled that cavity with extra amino acids. We therefore purchased a second clone of artemin with a C-terminal 6xHis tag (artemin-His) that would permit possible filling of the inner cavity while also addressing whether one could purify artemin using a tag on the C-terminus. We were able to successfully express and purify artemin-His with similar yields as Flag-artemin. Based on biochemical analyses (Supplementary Figures S1C,D), we obtained a fully assembled 24mer of artemin-His despite the tag being putatively localized to the interior of the complex. Cryo-EM analyses and image processing revealed certain differences between the N- and C-terminal tagged constructs. First, the central cavity of artemin-His appeared to be filled both in the micrographs and 2D class averages (Supplementary Figures S5A,B) as well as in the resulting 3D volume. This excess density relative to Flag-artemin is attributed to the 6xHis tag itself. Each Flag-artemin monomer had a 6xHis tag and a two amino acid linker, resulting in 192 extra residues getting packaged in the central cavity of a 24mer. (Supplementary Figures S5C,D). However, no refined density was observed in the 3D map at the very center suggesting a lack of any discernable secondary structure in the 6xHis tag. The final map obtained was at 2.56 Å (0.143 FSC; Supplementary Figures S5E,F) and was of lower resolution than the Flag-artemin map. A comparison of the C1 (no symmetry) *versus* octahedral symmetry map showed a minor disruption to the packing symmetry in the octahedral form which explains the lower resolution. Local refinement performed on the dimer density of artemin-His did not lead to any significant increase in resolution relative to the entire 24mer map suggesting that the small disruption of symmetry is already occurring at that base unit level. Alternatively, the disruption of the octahedral symmetry could be due to strain from excess packing and filling of the internal cavity but the different artemin maps do not show any change in outer dimensions. Since each voxel is less than 0.34 Å, the lack of a detectable change in outer diameter suggests any internal strain would be difficult to quantify.

We postulate that the disruption to symmetry packing of the artemin-His construct could be due to at least one of the C-terminal 6xHis tags being excluded from the inner cavity due to full packing of all the other tags and exiting the complex through either the 3-fold or 4-fold channels. This is supported by the observation that affinity purification of intact octahedral complexes using the His tag at the supposedly buried C-terminus was attainable at similar yields as Flag-artemin purification and near 90% of total expressed artemin. The 6xHis tag exclusion from the inner cavity could be due to electrostatic repulsion or stearic hindrance, or both. It is possible that even a few of these “flipped out” His tags would result in artemin-His 24mer being affinity purified on the Ni-NTA column. Alternatively, it is also possible that the 24mer exists in a rapid dynamic equilibrium with the dimer state in which case the dimer could be the dominant species getting selectively purified on the Ni-NTA column while the equilibrium shifts towards the 24mer after elution. Our biochemistry indicates that the majority of the artemin-His obtained post purification was a 24mer (Supplementary Figure S1) with no monomer or dimer band detected on the Native PAGE. Thus, it is difficult to assess biochemically whether the ability to purify the artemin-His 24mer is due to a flipped out or excluded His tag, or due to dimer/24mer dynamic equilibrium. To gain structural insight into this question, we decided to benchmark our workflow using commercially available apoferritin (Sigma Aldrich). Due to its homology to artemin as described above, and its use as a standard cryo-EM test specimen, we hypothesized that we could compare the number of particles needed to achieve a certain resolution for apoferritin and both artemin constructs as a semi-quantitative measure of symmetry stability. A 1 mg/ml solution of apoferritin was loaded on holey carbon grids, blotted for 2 s and plunge frozen for cryo-EM data collection. Movies were collected at similar pixel size and all image processing was performed similar to that for Flag-artemin and artemin-His. At 160 k particles, the reconstruction of apoferritin hit a resolution of 2.05 Å which matches well with the 2.04 Å map of Flag-artemin using a similar 167 k particles. This indicates that Flag-artemin 24mer had near ideal octahedral symmetry as it tracked with expected resolution based on particle number. In comparison, when the commercially purchased apoferritin dataset used all 674 k particles of the refined dataset it resulted in an improved map at a resolution of 1.91 Å (Supplementary Figure S6). This trend of more particles resulting in higher resolution for apoferritin is well characterized in the cryo-EM field (Yip et al., 2020). Interestingly, while the artemin-His dataset has 4x as many particles as the Flag-artemin dataset and an equivalent number of particles as the full apoferritin dataset, the artemin-His map yielded significantly worse resolution (2.56 Å). Taken together, the above data indicate that artemin-His experiences disrupted symmetry compared to Flag-artemin, but we also wanted to evaluate these samples with native MS to determine the 6xHis tag effect on stability.

**FIGURE 4:**
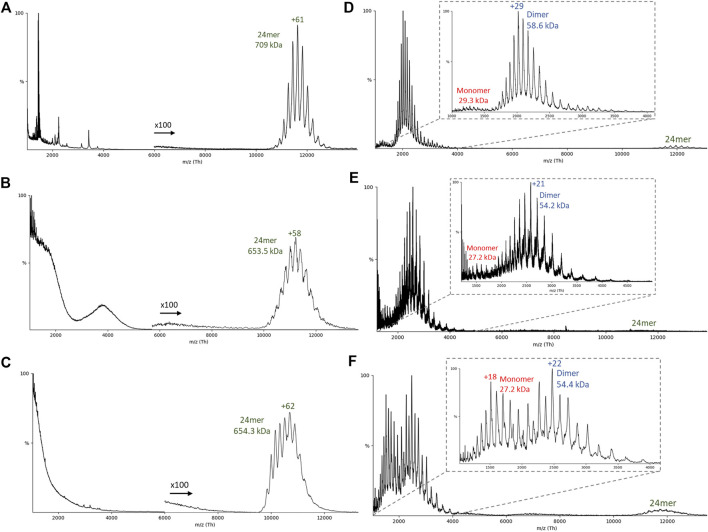
Native mass spectrometry of artemin constructs. **(A–C)** Representative native MS spectrum and the corresponding **(D–F)** collision induced dissociation (CID) spectrum of the resulting released monomers/dimers (A/D) Flag-artemin, (B/E) artemin-His, and (C/F) Fluorescent artemin-His.

Using native MS, the mass of the intact 24mer for Flag-artemin was observed to be 709 kDa (theoretical: 696 kDa) while artemin-His was 653.5 kDa (theoretical: 650 kDa) (Figures 4A–C). After isolating the 24mer, collision-induced dissociation (CID) was used to release smaller subunits (Figures 4D,E). In CID, the protein ions are accelerated into a pressurized collision cell where the protein ions then collide with a neutral gas (argon in this experiment). As the number of collisions increase, the internal energy of the protein increases as well, causing potential unfolding and release of smaller subunits and/or bound ligands (McLuckey, 1992; Benesch, 2009). Typically, a monomer is expected to be stripped from the complex during CID. For both Flag-artemin (Figure 4D) and artemin-His (Figure 4E), only a small population of monomers was observed, but the predominant species was dimers. This unusual CID behavior is consistent with the observed inter-subunit disulfide linkages in the cryo-EM structure (Figure 2C) that help stabilize dimeric interactions. Interestingly, the relative amount of monomers for artemin-His is increased and the 24mer peaks are less well resolved compared to the Flag-artemin. This may be indicative of lower stability for artemin-His but is not definitive. To further disrupt the stability of artemin-His, we doped fluorescent lysine tRNA into the cell-free reaction in hopes this approach might further stress the complex assembly or dimer stability due to small steric hindrance. The use of doping rather than complete swapping of all lysine tRNA permitted the random incorporation of fluorescently labelled lysines into the artemin monomer. This was important since there are 15 lysines in the full-length 230 amino acid sequence of artemin (excluding tags) with one lysine being immediately adjacent to Cys61 involved in disulfide bonding and several in loop L’ and at the C-terminus. The fluorescent artemin-His (Fluor artemin-His) complex expressed and purified like artemin-His and was found to be a clean octahedral complex by Native PAGE and was detected as a 24-mer by native MS at 654.3 kDa (Figure 4C). Interestingly, when CID was performed with the same settings as used above for Flag-artemin and artemin-His, monomeric and dimeric species were released from Fluor artemin-His (Figure 4F) at nearly equivalent levels. The masses of the released monomers and dimers in the Fluor artemin-His were essentially the same as those in the artemin-His, although the Fluor artemin-His dimer showed a difference of around 200 Da which could correspond to the presence of 1 fluorescent lysine modification (addition of 1 bodipy molecule) per dimer. Unfortunately, all of the detected mass difference is within experimental uncertainty, and therefore cannot precisely quantify the number of incorporated fluorescent tags. Overall, the similar masses between the labeled and unlabeled proteins suggested a low incorporation rate of the Fluor tag. However, despite the low fluorescent lysine incorporation rate, the increased presence of detectable monomeric species for the Fluor artemin-His sample suggests the limited modifications indirectly disrupted part of the dimeric substructure, possibly *via* prevention of inter-subunit disulfides. Additionally, Fluor artemin-His showed higher charge state distributions than the other artemin 24mers (Figures 4C *versus* Figures 4A,B) and appeared to be less symmetric, suggesting multiple overlapping distributions (a more distinct bimodal distribution from another replicate is shown in Supplementary Figure S7). It is generally accepted that charge state distribution correlates with protein conformation, although the detailed mechanisms are under debate (Grandori, 2003; Hall and Robinson, 2012). The higher charge state of Fluor artemin-His implies a less compact structure that is potentially due to further disruption of the artemin interfaces. Therefore, the change of charge state distributions for the different complexes suggests structural changes in response to terminal and lysine tagging with the unmodified Flag-artemin being the most stable 24mer.

## 4 Discussion

Here we describe a method that allowed us to progress from receiving a custom synthesized gene/plasmid of artemin, through expression, purification, and cryo-EM structure determination at sub 2.5 Å resolution within 2 weeks. This is also the first report of an experimental structure for the diapause chaperone artemin, almost 40 years after it was first discovered. We found that the C-terminal region important for chaperoning is positioned differently than all prior homology modeling, molecular dynamics and even recent Alphafold2 and RosettaFold models suggest. The C-terminal loop L’ and helix F were observed to provide additional interfaces for artemin dimers to interact and stabilize the 24mer assembly. These results raise new questions regarding the structural details of how artemin functions as a chaperone. For example, the functional chaperone unit of artemin is believed to be the dimer form, but does it retain the same overall fold as the dimer in the 24mer or does the C-terminal region (or other regions) refold during chaperoning? A logical extension of our study would be structural studies of artemin “caught in the act” of chaperoning a target protein like citrate synthase or lysozyme or bound to RNA. While an artemin monomer or dimer on its own would be difficult to resolve using single particle cryo-EM, a dimer interacting with the chaperoned target would be big enough for cryo-EM studies if the binding interfaces between artemin dimer and target are specific. Native MS methods such as collision induced unfolding (Dixit et al., 2018) and variable temperature (Laganowsky et al., 2021) electrospray will also provide unique contributions to the biophysical characterization of the stability and dynamics of these assemblies. With the structure of artemin now in hand, these possible future studies should help illuminate the unique holdase-like characteristics and mechanisms employed by artemin during its protein and RNA chaperoning activities.

## Data Availability

The datasets presented in this study can be found in online repositories. The names of the repository/repositories and accession number(s) can be found in the article/Supplementary Material.
