# Supplementary material for: Cryo-EM structure of the diapause chaperone artemin
Source: Front Mol Biosci. 2022 Nov 28;9:998562. doi: 10.3389/fmolb.2022.998562 (PMC9742257; doi:10.3389/fmolb.2022.998562)
Supplement: Supplementary file 1 [file DataSheet1.docx]

Supplementary Material

Cryo-EM structure of the diapause chaperone artemin

**Amar D. Parvate^1^, Samantha M. Powell^1^, Jory T. Brookreson^1^, Trevor H. Moser^1^, Irina V. Novikova^1^, Mowei Zhou^1^ and James E. Evans^1,2^**

^1^ Pacific Northwest National Laboratory, Environmental Molecular Sciences Laboratory, Richland, WA, USA

^2^ Washington State University Pullman, School of Biological Sciences, Pullman, WA, USA

*** Correspondence:**Corresponding Author
[James.Evans@pnnl.gov](mailto:James.Evans@pnnl.gov)

# Supplementary Figures and Tables


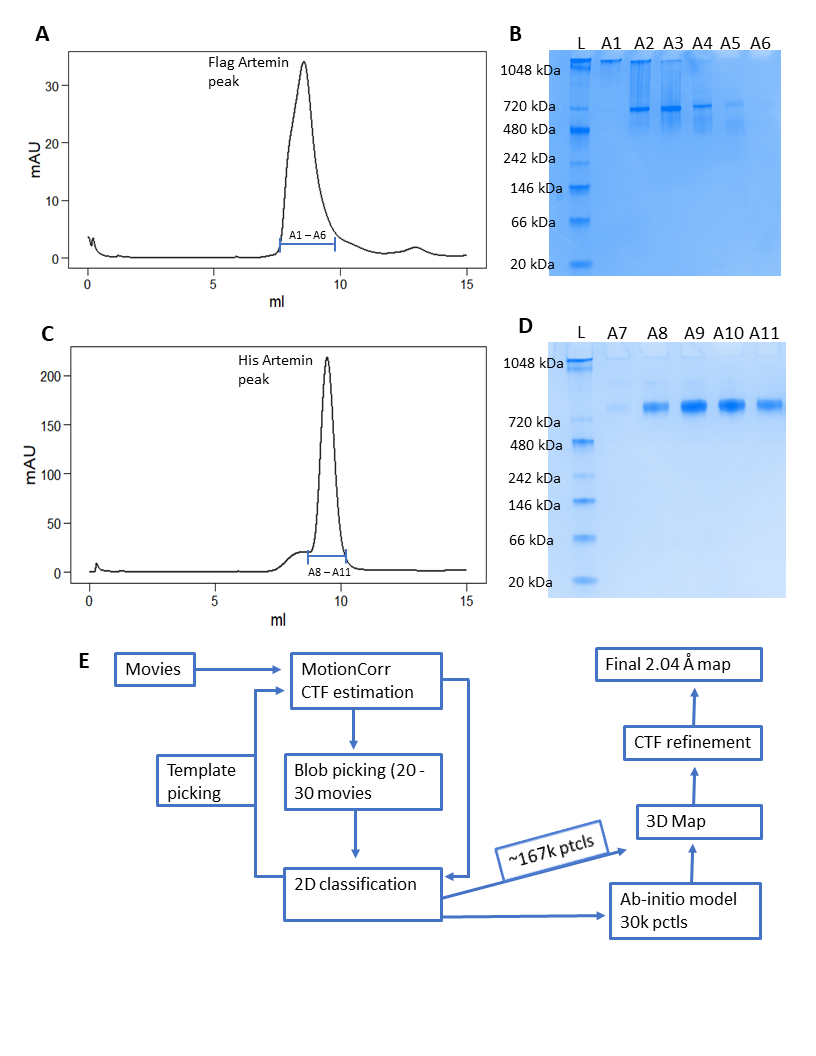


**Supplementary Figure 1** – **Biochemistry and image processing workflow for Flag-artemin.** A) FPLC trace of artemin purified over a Superdex 200 column with a single homogenous peak for artemin. Gel B) Native PAGE analyses of fractions A1-A6 from the FPLC. Distinct clear bands near ~720 kDa mark indicate a 24mer of Flag-artemin. C) FPLC trace of artemin purified by SEC with a single homogenous peak for artemin. D) Native PAGE analyses of fractions A7-A11 from the FPLC. Distinct clear bands near ~720 kDa mark indicate a 24mer of Flag-artemin. L = lane for NativeMark Protein Ladder. E) Image processing workflow implemented using cryoSPARC and cryoSPARC Live.

**
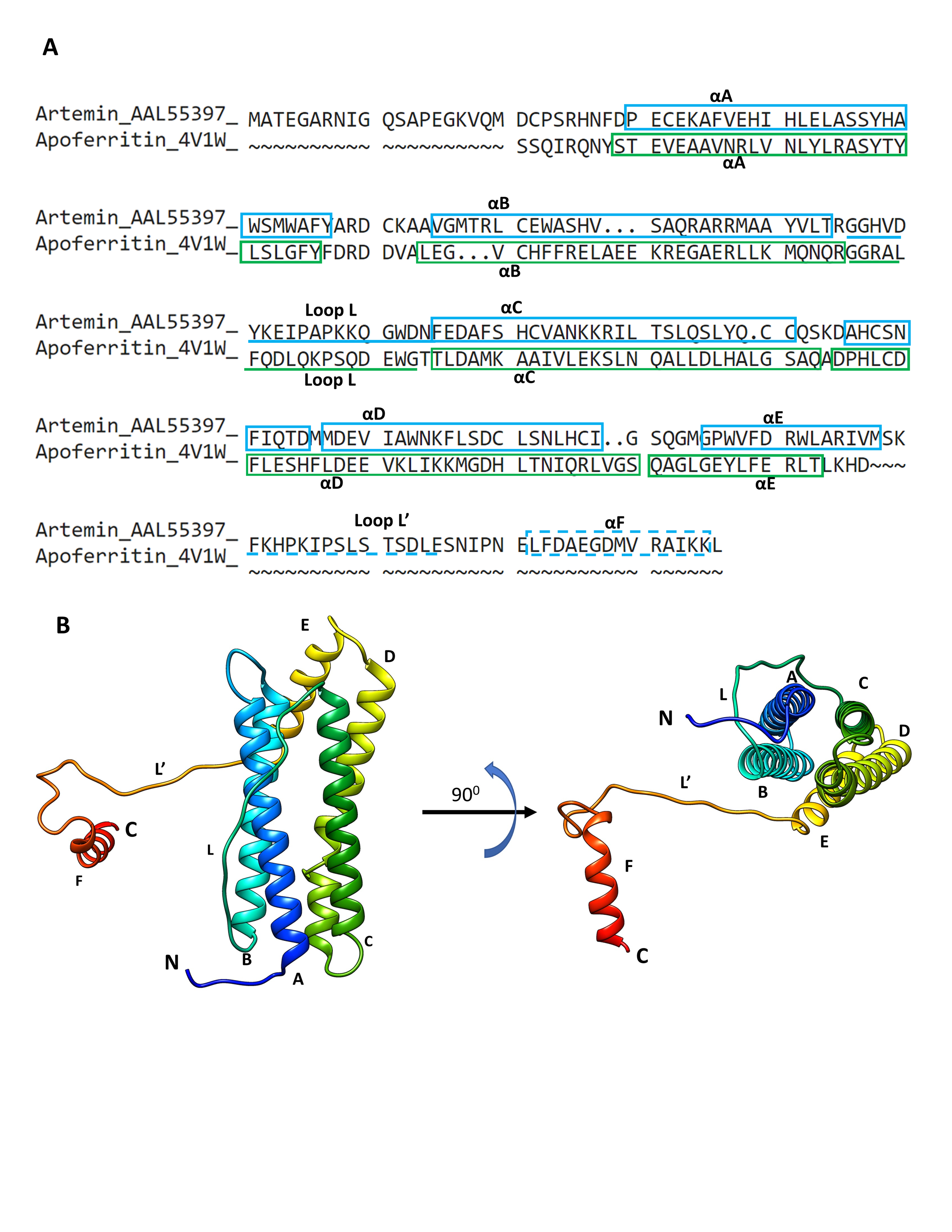
Supplementary Figure 2 – Secondary structure-based sequence comparison between artemin and apoferritin.** A) Primary sequences of artemin and apoferritin (4V1W) are aligned and the known alpha helical regions and loops are compared regions. Artemin has 6 α-helices (A-F, blue border) and 2 Loops (L, L’ blue line) relative to 5 α-helices (A-E, green boundary) and 1 loop (green line). The Loop L’ and Helix F in artemin indicated in dashed lines have no comparative region in apoferritin. B) N to C terminal rainbow depiction of the final model of an artemin monomer.


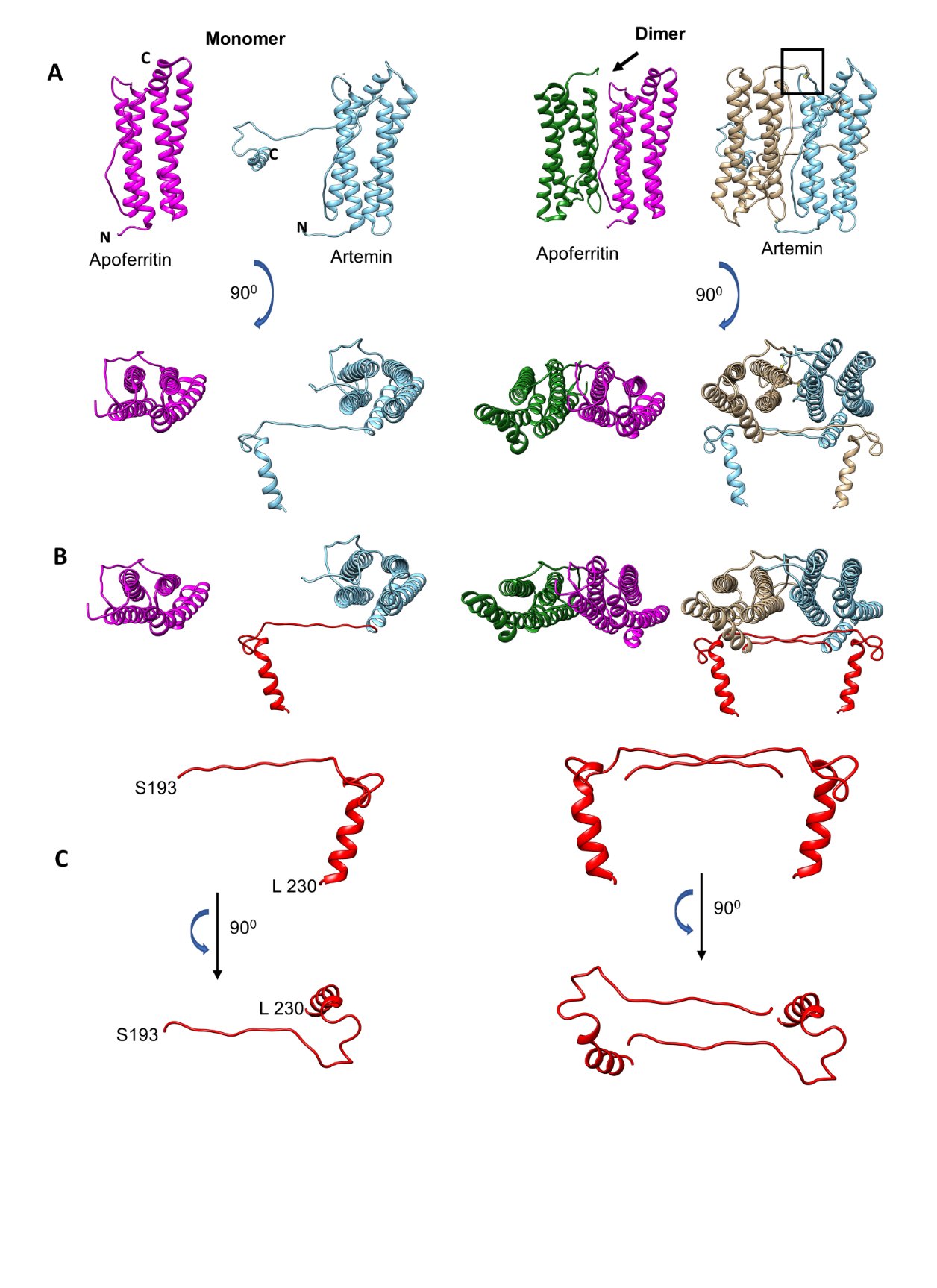


Supplementary Figure 3 - Comparison of apoferritin vs artemin monomer and dimer. Two anti-parallel monomers make up a dimer of apoferritin (magenta and green) and artemin (blue and tan). A) Equivalent dimers and monomers of apoferritin and artemin are visualized in different orientations for comparison. The black box indicates the C22-C61 disulfide bond in the apoferritin monomer whereas the black arrow indicates an equivalent position in apoferritin where no disulfide bond is present. B) The red colored fragment indicates the region in artemin (aa 193-230) for which no corresponding fragment is present in apoferritin. C) The secondary structure from aa 193-230 from an artemin monomer and dimer is presented slightly magnified and in two orientations.


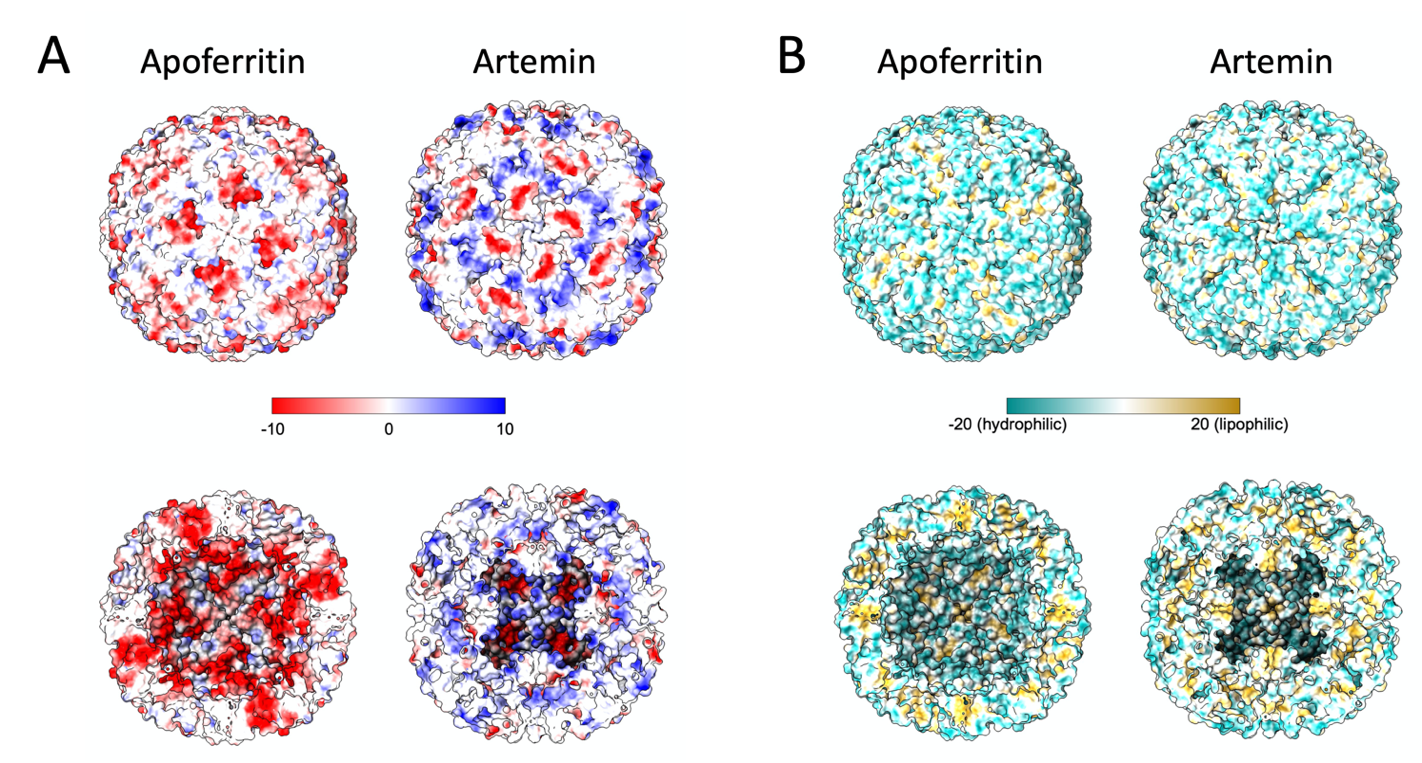


**Supplementary Figure 4 – Comparing electrostatic and coulombic surface maps of apoferritin and artemin.** A) Electrostatic charges visualized on the overall map of apoferritin and artemin (scale -10 to 10) as visualized in UCSF ChimeraX. Artemin has relatively more positive (blue) charge on surfaces exposed to the solvent externally and internally. B) Coulombic (hydrophobicity) map of apoferritin and artemin (scale -20 to 20) as visualized in UCSF ChimeraX. For both panels, the top images show the outer surface map while the bottom images show the internal view cutting halfway through the assembled 24mer. Artemin has more hydrophilic residues (cyan) exposed on the surface. All surface rendering views aligned along the C4 axis.

**
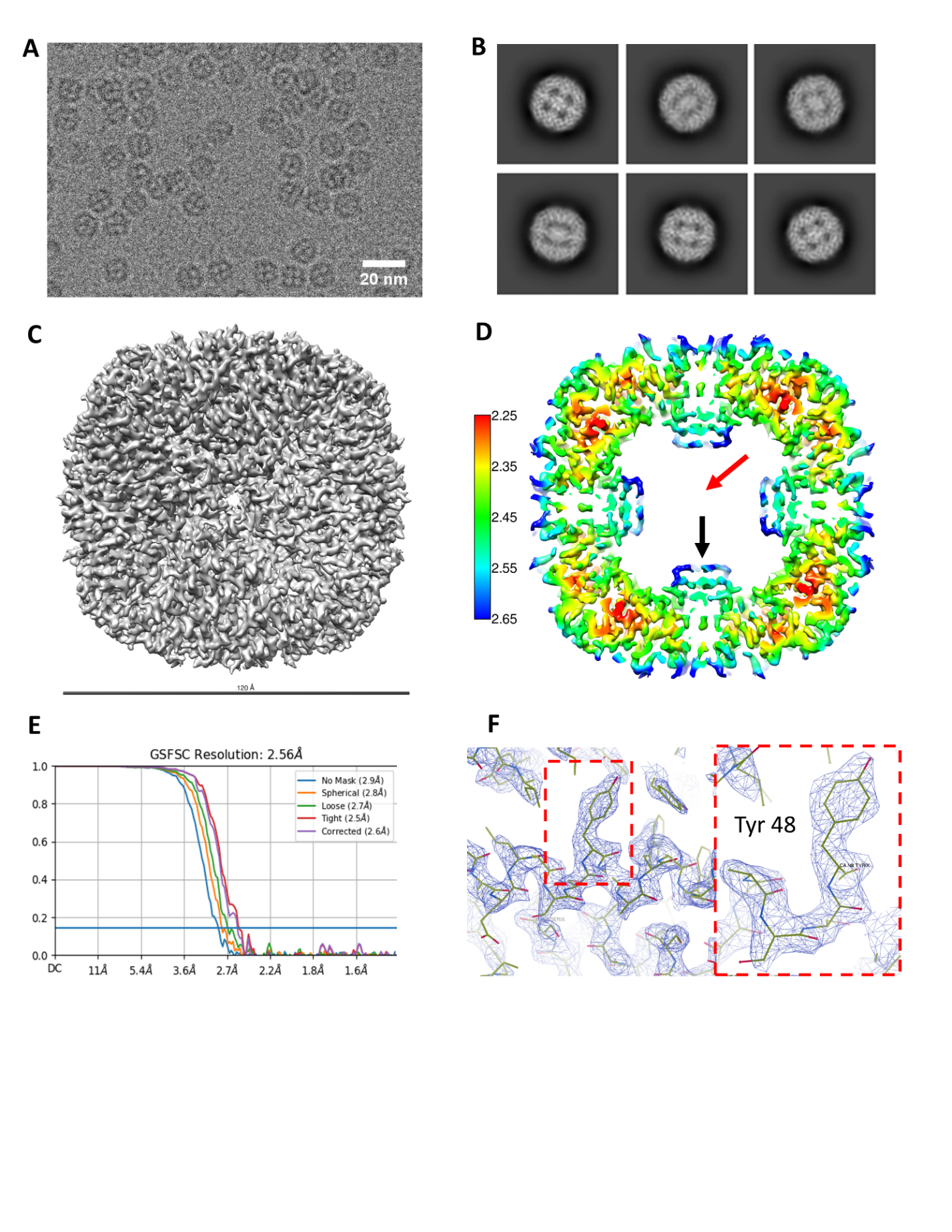
Supplementary Figure 5** - Data processing results for artemin-His. A) Representative micrograph of artemin-His showing the central cavity is distinctly filled with density attributed to 6xHis tags on each monomer. B) 2D classes showing the C-terminal of the monomer does point inwards from the shell, and fully fills the central cavity. C) Cryo-EM map of artemin-His with ~120 Å diameter D) Thin virtual slice through a resolution heat map showing the C-terminal alpha helices pointing inwards (black arrow) into the cavity. Central cavity does not show any density for His tags corresponding to the 2D classes or micrographs (red arrow). Scalebar indicates resolution in Å. E) Resolution estimated by gold standard at 2.56 Å at 0.143 FSC. F) Quality of the map as inspected by fitting of alpha helices.


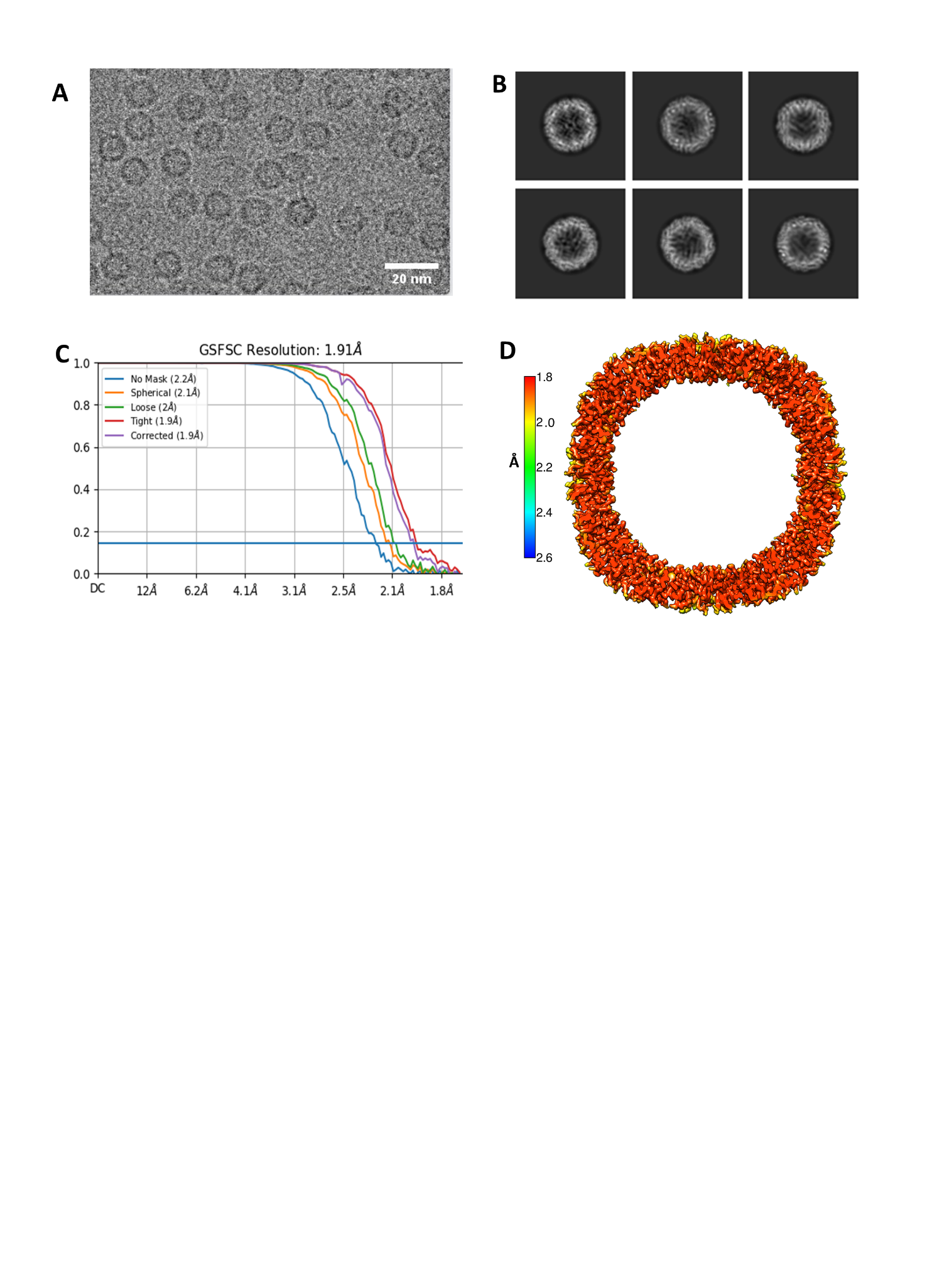


Supplementary Figure 6 - Image processing and cryo-EM map of commercially purchased apoferritin – A) Representative image of apoferritin showing monodisperse apoferritin particles almost in a monolayer. B) Representative 2D classes obtained showing various orientations of apoferritin. C) A 1.91 Å resolution map apoferritin at 0.143 FSC (gold standard) D) Virtual slice through a local resolution heat map of apoferritin showing various regions of the map resolved between 1.8 and 2.0 Å.


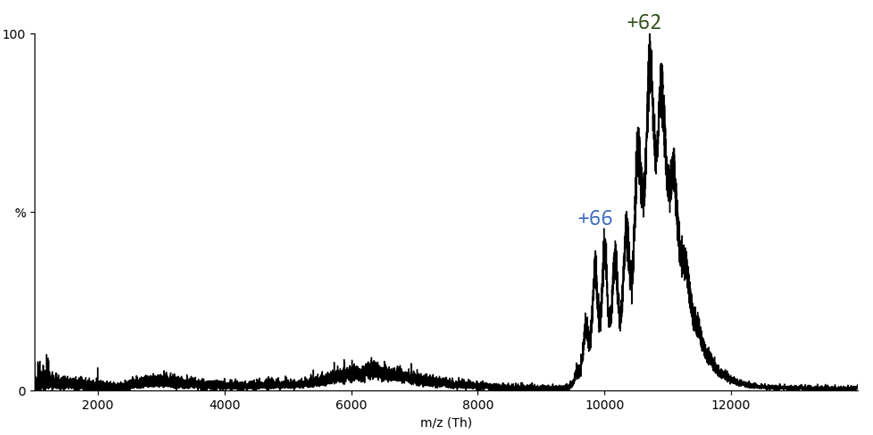


**Supplemental Figure 7 – Unfolding of fluorescent artemin-His.** Native MS shows two distinct charge state populations centered around +66 and +62, indicating the multiple conformations (potentially less compact at +66) of Fluor artemin-His.

**Supplement Table 1 – Amino acid sequence for all clones**

| **artemin-His** | MATEGARNIGQSAPEGKVQMDCPSRHNFDPECEKAFVEHIHLELASSYHAWSMWAFYARDCKAAVGMTRLCEWASHVSAQRARRMAAYVLTRGGHVDYKEIPAPKKQGWDNFEDAFSHCVANKKRILTSLQSLYQCCQSKDAHCSNFIQTDMMDEVIAWNKFLSDCLSNLHCIGSQGMGPWVFDRWLARIVMSKFKHPKIPSLSTSDLESNIPNELFDAEGDMVRAIKKL**GSHHHHHH** |
| --- | --- |
| **Flag-artemin** | **MDYKDHDGDYKDHDIDYKDDDDKL**ATEGARNIGQSAPEGKVQMDCPSRHNFDPECEKAFVEHIHLELASSYHAWSMWAFYARDCKAAVGMTRLCEWASHVSAQRARRMAAYVLTRGGHVDYKEIPAPKKQGWDNFEDAFSHCVANKKRILTSLQSLYQCCQSKDAHCSNFIQTDMMDEVIAWNKFLSDCLSNLHCIGSQGMGPWVFDRWLARIVMSKFKHPKIPSLSTSDLESNIPNELFDAEGDMVRAIKKL |

- Sequences for affinity tags (6xHis and 3xFlag) along with spacers are shown bold

and underlined

**Supplement Table 2 – Dataset details for all artemin constructs**

| **Dataset /**  **EMDB ID** | **artemin-His**  **EMD-24707** | **Flag-artemin**  **EMD-24706** | **Sigma Apoferritin**  **EMD-24145** |
| --- | --- | --- | --- |
| Magnification | 130,000 | 130,000 | 215,000 x |
| Super resolution | Yes | Yes | No |
| Pixel size (Å) | 0.3398 | 0.3398 | 0.4108 |
| Total dose (e^-^/ Å^2^) | 52 | 41 | 40 |
| Defocus range (µm) | -0.3 to -1.3 | -0.3 to -1.3 | -0.3 to -1.3 |
| Micrographs collected | 6578 | 6626 | 11028 |
| Micrographs utilized | 2494 | 4933 | 9583 |
| Initial particles no. | 792,493 | 964,141 | 733,858 |
| Final particle no. | 660,454 | 167,408 | 674,864 |
| Symmetry imposed | Octahedral | Octahedral | Octahedral |
| Resolution (0.143 FSC) | 2.58 Å | 2.04 Å | 1.91 |
| Concentration loaded | 1 mg/ml | 1.5 mg/ml | 1 mg/ml |

**Supplement Table 3 – Model validation statistics for artemin 24mer (PDB: 7RVB)**

| **Composition** | |
| --- | --- |
| Chains | 24 |
| Atoms | 40224 (Hydrogens: 0) |
| Residues | Protein: 5016, Nucleotide: 0 |
| Water | 0 |
| Ligands | 0 |
| **Bonds (RMSD)** | |
| Length (Å) (# > 4s) | 0.004 (0) |
| Angles (°) (# > 4s) | 0.543 (3) |
| Molprobity score | 1.12 |
| Clashscore | 1.94 |
| **Ramachandran plot (%)** | |
| Outliers | 0.00 |
| Allowed | 2.90 |
| Favored | 97.10 |
| Rotamer outliers (%) | 0.55 |
| Cß outliers (%) | 0.00 |
| **Model vs. Data** |  |
| CC (volume) | 0.91 |

**Supplement Table 4 – Helices and loops of artemin based on similarity to apoferritin helices**

| **Residue number** | **Description** |
| --- | --- |
| 30-57 | Helix A |
| 65-91 | Helix B |
| 92-111 | Loop L |
| 112-137 | Helix C |
| 142-173 | Helix D |
| 179-192 | Helix E |
| 193-208 | Loop L’ (not present in apoferritin) |
| 216-229 | Helix F (not present in apoferritin) |
